# Direct Observation of Membrane Insertion by Enveloped Virus Matrix Proteins by Phosphate Displacement

**DOI:** 10.1371/journal.pone.0057916

**Published:** 2013-02-28

**Authors:** Benjamin W. Neuman, Gabriella Kiss, Hawaa M. N. Al-Mulla, Terje Dokland, Michael J. Buchmeier, Thomas Weikl, David Schley

**Affiliations:** 1 School of Biological Sciences, University of Reading, Reading, Berkshire, United Kingdom; 2 School of Medicine, Emory University, Atlanta, Georgia, United States of America; 3 Department of Microbiology, University of Alabama, Birmingham, Alabama, United States of America; 4 Department of Medicine and Department of Molecular Biology and Biochemistry, University of California Irvine, Irvine, California, United States of America; 5 Department of Theory and Bio-Systems, Max Planck Institute of Colloids and Interfaces, Potsdam, Germany; 6 The Pirbright Institute, Pirbright, Surrey, United Kingdom; Centre National de la Recherche Scientifique, Aix-Marseille Université, France

## Abstract

Enveloped virus release is driven by poorly understood proteins that are functional analogs of the coat protein assemblies that mediate intracellular vesicle trafficking. We used differential electron density mapping to detect membrane integration by membrane-bending proteins from five virus families. This demonstrates that virus matrix proteins replace an unexpectedly large portion of the lipid content of the inner membrane face, a generalized feature likely to play a role in reshaping cellular membranes.

## Introduction

Life as we know it comes wrapped in membranes. These semipermeable barriers are necessary to maintain the electrical and chemical gradients essential to life. In eukaryotes, intracellular vesicle transport is mediated by vesicle transport proteins that are needed to move cargo between organelles and across the plasma membrane [1], [2]. The best studied examples of vesicle-forming proteins appear to initiate membrane curvature by inserting amphipathic protein domains into one side of the membrane, displacing lipid molecules and effectively stretching one membrane face more than the other [3]–[5]. While there is considerable evidence for this mechanism, it has proved difficult to directly demonstrate that lipid molecules are displaced when proteins are inserted, as stipulated by the model.

Enveloped viruses encode matrix proteins that mold the membrane around new virus particles as they exit a host cell, a process analogous to vesicle transport. Matrix proteins occupy a middle position inside the virus between the membrane and the virus core, are essential for virus assembly, and some direct the release of virus-like particles without help from other viral proteins [6]. However, the matrix-membrane interaction remains poorly understood because of the technical difficulty of directly investigating processes that occur within the membrane.

## Results

**Figure 1:**
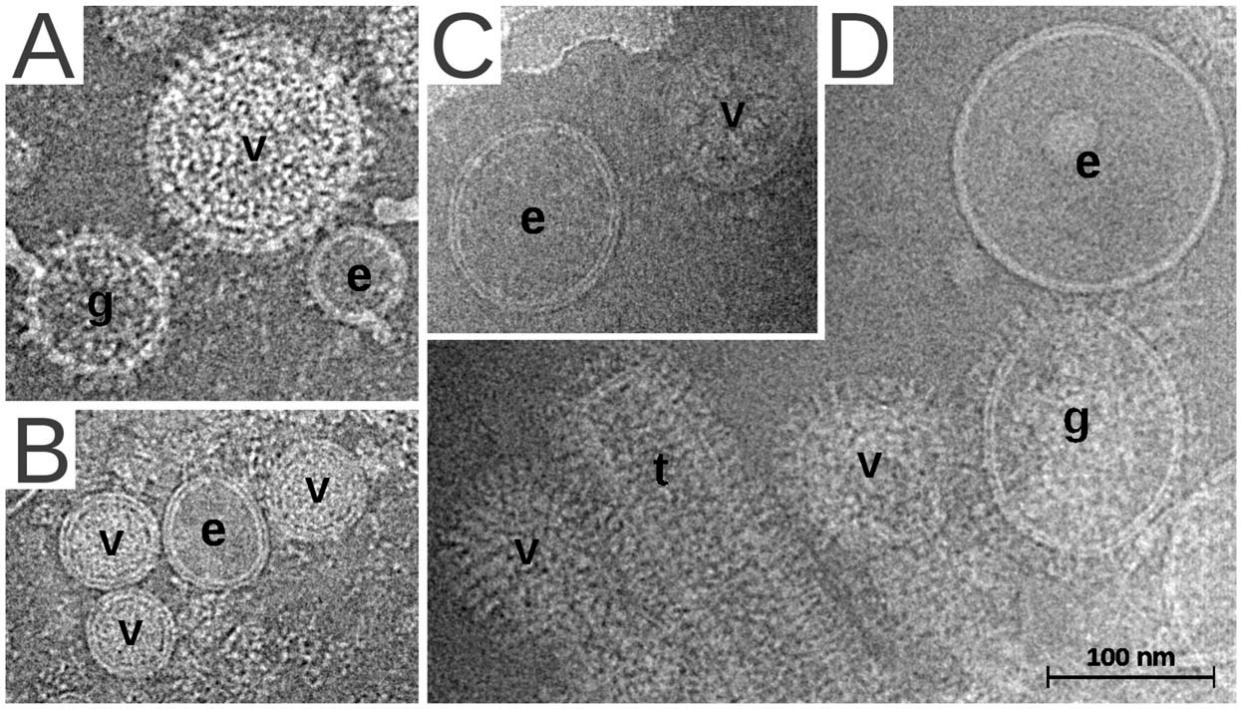
Cryo-electron micrographs of virus preparations. The images include virus particles (v), GP vesicles (g), empty vesicles (e) and tubular hollow particles (t). Preparations of *Tacaribe virus* (A), *Porcine respiratory and reproductive syndrome virus* (B), *Severe acute respiratory syndrome coronavirus* (C) and *Influenza A virus* (D) are shown to illustrate the double-ringed appearance of the membrane.

In order to see how working matrix proteins interact with the membrane, we used cryo-electron microscopy to take pictures of virus populations in a near-native environment. In addition to viruses, these populations naturally contained a few empty vesicles and a heterogeneous collection of incompletely assembled viruses that were released from the same cells as the virus. Of these, the most interesting were called GP vesicles, which had virus surface glycoproteins but lacked a visible matrix (GP vesicles; Fig. 1).

**Figure 2:**
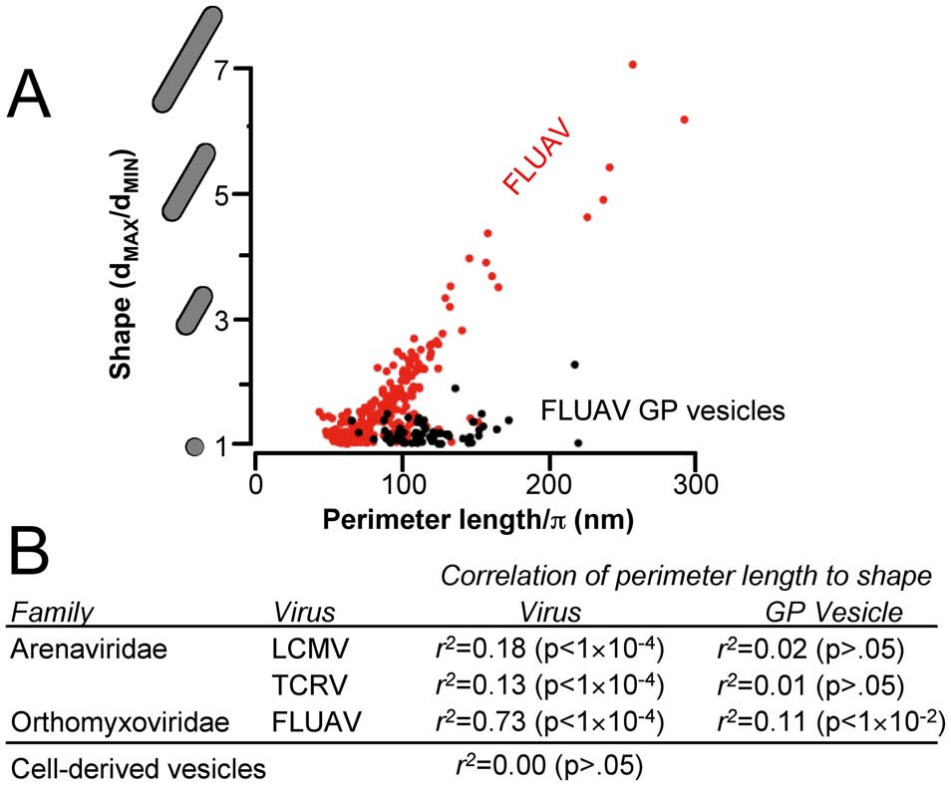
Presence of matrix proteins at the virion surface is necessary to maintain elongated virus shapes. (A) Shape and perimeter length are shown for 252 *Influenza A virus* particles and 66 GP vesicles. Coefficients of determination and statistical confidence measures are shown for virus particles and GP vesicles of *Lymphocytic choriomeningitis virus* (LCMV), *Tacaribe virus* (TCRV) and *Influenza A virus* (FLUAV), and for empty vesicles of cellular origin (B).

Viruses, GP vesicles and empty vesicles were measured to determine whether the shape of the virus membrane was altered in the presence of matrix proteins. Virus-sized unilamellar vesicles in these micrographs were generally spherical as expected [7], with an average ratio of 1.05±0.10 for the largest to the smallest visible diameter. Three of the eleven viruses studied showed a statistically significant correlation between virus size and shape, ranging from small round viruses to large sausage-shaped particles with maximum diameters up to seven times as long as the shortest diameter (Fig. 2A), demonstrating that membrane shape is altered when virus proteins are present. The viruses selected here are all relatively simple, in that matrix proteins and the transmembrane anchors of the surface glycorproteins are the only virus components in direct contact with the membrane. The correlation between size and shape was absent or reduced for GP vesicles (Fig. 2B), demonstrating that the matrix proteins of these viruses are necessary for membrane bending, as reported previously [8].

We then investigated whether any of these virus matrix proteins could integrate into the membrane and replace lipid molecules, following the mechanism proposed to explain how Sar1p [3], Epsins [4] and Influenza A virus M2 [5] induce membrane curvature. We looked for differences in the apparent brightness of the membrane in electron micrographs as a way to detect changes in the amount protein in each side of the membrane. This method works on the principle that electrons passing through a sample are scattered approximately in proportion to the square of the atomic number of the constituent atoms [9]. Therefore, when a substance made of small atoms such as protein is integrated into the membrane, membrane lipids are displaced, making that membrane face appear correspondingly dimmer compared to cell-derived vesicle controls of similar size in the same image.

Matrix proteins from different viruses are believed to use different methods to interact with the membrane. Our dataset included representatives of the three common membrane anchor types, including viruses with transmembrane matrix proteins (*Coronaviridae* M, *Arteriviridae* M and GP5), acylated matrix proteins (*Retroviridae* Gag, *Arenaviridae* Z) and non-acylated proteins (*Orthomyxoviridae* M1). The three-dimensional structures of these proteins, where known, appear to be unrelated [10]–[12].

We compared transects through the edge of viruses, vesicles and GP vesicles, where the phosphorous atoms of membrane lipids align to give the membrane a double-ringed appearance (Fig. 1). Viruses were compared to vesicles using a linear mixed-effects model in order to detect the replacement of membrane phospholipid by lower electron density material such as protein. Similarly, GP vesicles were compared to vesicles to distinguish the effects of matrix proteins from other viral membrane proteins.

**Figure 3:**
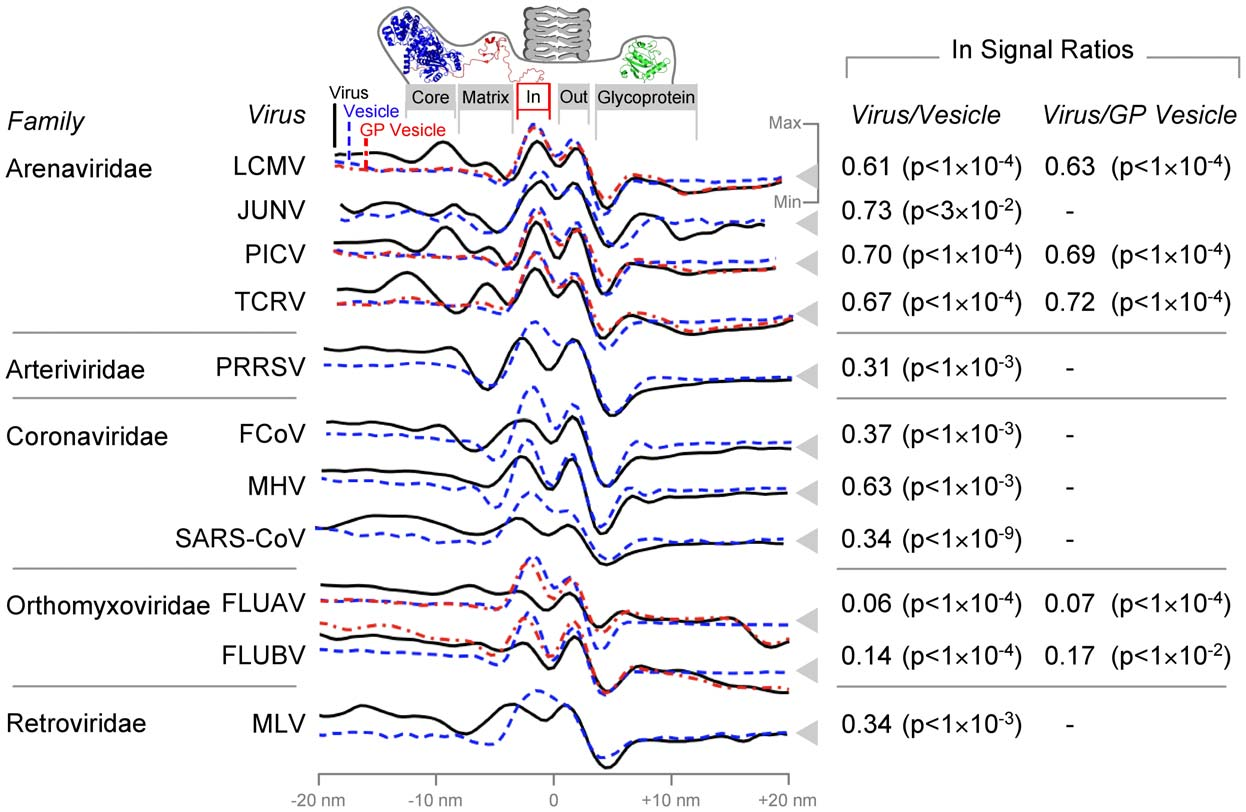
Lipid phosphate is displaced in the presence of viral matrix proteins. Relative electron microscope signal intensity is shown on the vertical axis with average background intensity marked by a gray triangle. The horizontal axis represents radial distance from the midpoint of the membrane. The boundaries of the inner (In) and outer (Out) membrane phosphate rings measured in this study are shown for viruses (black), empty vesicles (blue) and virus-like particles that contain surface glycoproteins but lack a visible matrix layer (GP vesicles; red). Approximate positions of the nucleoprotein (core; 3MX5), matrix (2KO5) and glycoprotein (3KAS) structures in arenavirus particles are shown as a reference. P-values relate to comparison of inner phosphate ring signals with viruses as described in the Methods section. Comparisons are omitted where GP vesicles were not available. Virus names are abbreviated as follows: *Lymphocytic choriomeningitis virus* (LCMV), *Junin virus* (JUNV), *Pichinde virus* (PICV), *Tacaribe virus* (TCRV), *Porcine respiratory and reproductive syndrome virus* (PRRSV), *Feline coronavirus* (FCoV), *Mouse hepatitis virus* (MHV), *Severe acute respiratory syndrome coronavirus* (SARS-CoV), *Influenza A virus* (FLUAV), *Influenza B virus* (FLUBV), *Murine leukaemia virus* (MLV).

The intensity of the virus inner phosphate rings differed strongly from internal vesicle controls, with a significantly lower signal in all 11 viruses considered (Fig. 3). The inner phosphate ring (area marked In) in native virus particles was only 45±23% (blue vs. black, n = 11) that of the vesicles and 46±31% (red vs. black, n = 5) that of GP vesicles, demonstrating that the reduction in inner leaflet phosphate is matrix protein dependent, and not solely due to transmembrane surface glycoproteins. To our knowledge, this is the first time lipid displacement has been tracked to demonstrate membrane protein integration.

**Figure 4:**
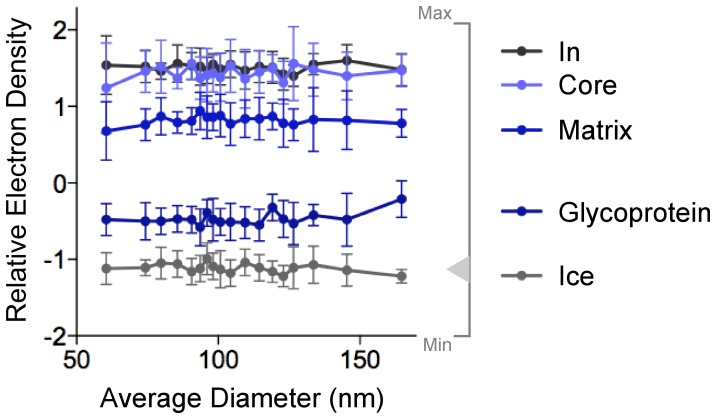
Apparent electron density is constant for small and large virus particles. The virion edge was sampled at four positions described in Fig. 3 (In, Core, Matrix, Glycoprotein) and at the background ice beyond the Glycoprotein (Ice). Each datapoint shows the average density for 8 samples from 12 *Tacaribe virus* particles of similar size. Error bars indicate standard deviation.

We had two main concerns regarding phosphate displacement. The first was mechanical – as the virus gets smaller, the membrane curves more per unit area. Large and small particles differ in curvature per unit length, which could change how many lipid molecules the electron beam encounters at the particle edge, causing large particles to appear brighter than small particles. We tested for this effect by examining the intensity of internal, membrane and external features of 216 *Tacaribe virus* particles, which ranged from 40 nm to 260 nm in diameter (Fig. 4). Image intensity did not vary noticeably over the virus size range, demonstrating that curvature does not have a noticeable effect on intensity for virus-sized membranes.

**Figure 5:**
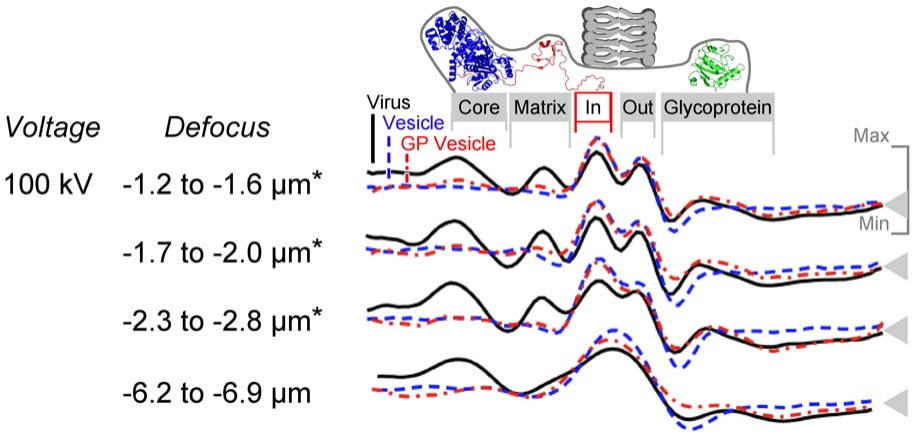
Phosphate signal dimming effects are visible over a wide range of focal distance. Average electron density is shown for images of *Tacaribe virus* particles, GP vesicles and associated vesicles that were recorded as defocus series. Data marked with an asterisk contributed to the TCRV line in Fig. 3.

Secondly, we considered whether electron interference at the matrix-membrane interface could explain the decreased inner membrane phosphate signal. Contrast in electron microscopy images has two main sources, called amplitude and phase contrast. Amplitude contrast occurs when electrons collide with atoms in the sample and are scattered, and changes with the atomic mass of the sample [13]. Phase contrast is caused by electron interference, and changes with defocus [13]. To examine the effects of phase contrast, TCRV images were grouped according to defocus and analysed as before. Matrix-dependent phosphate displacement was observed across the defocus range (Fig. 5), though the effect was strongest in images where both phosphate rings were distinctly visible. We concluded that matrix-dependent displacement was not likely caused by electron interference.

## Discussion

The presence of transmembrane helixes the virus glycoproteins studied here would be expected to displace less than 10% of the membrane lipid, based on helix diameter, the number of helices per glycoprotein complex and the spacing between complexes. The inner leaflet phosphate displacement by matrix protein was approximately five-fold greater than the displacement of viral glycoproteins. This adds *Arenaviridae* Z, *Orthomyxoviridae* M1, *Coronaviridae* M, *Arteriviridae* M and GP5 and *Retroviridae* Gag, to the expanding list of curvature activating proteins that are inserted into one face of the membrane.

One effect of protein insertion would be to push lipids aside, temporarily stretching one membrane face to create an inward bulge around the budding virus. The line tension that would be created around the inserted matrix proteins could then be resolved by squeezing the new virus particle outward, a mechanism explored mathematically to explain how vesicular transport proteins work [14].

Other methods that have been used to probe protein-membrane interactions include neutron diffraction [15] and more recently, electron spin resonance [16], [17]. Electron spin resonance can be used to map and understand the kinetics of interactions between specific amino acids and lipid molecules [18]. However, neither provides a convenient way to measure membrane curvature where the protein contacts the membrane, and both analyze the entire sample at once, making it difficult to interpret heterogeneous samples such as native enveloped viruses. Electron spin resonance also requires spin-labeling, which can be difficult to perform without perturbing fragile membrane interactions. Phosphate displacement analysis can be used on native samples, and it provides complementary data on the location and footprint of single protein-membrane interactions from within the visual context of a cryo-electron micrograph.

Phosphate displacement analysis is therefore well suited to directly test hypotheses about how membrane interactions are related to protein conformation and membrane curvature [3]–[5], [19], and should be useful for understanding rapidly changing and heterogeneous samples. Phosphate displacement analysis does not require any specialized reagents or instruments beyond access to a standard cryo-electron microscopy suite, and is potentially inexpensive because it can be performed on archival micrographs.

The phosphate displacement analysis presented here is a powerful technique that can be used to explore the process of curvature induction and identify new membrane-remodeling proteins. The diversity of these viral and cellular vesicle forming proteins is remarkable, but this extreme example of convergent evolution hints at the wider possibilities for natural and directed membrane manipulation. The prevalence of unifacial membrane integration, which has evolved to work from a wide variety of structural contexts, suggests that these proteins may be useful models for synthetic biology applications that require precise control of the membrane.

## Methods

### Viruses and Cells

Lymphocytic choriomeningitis virus-Arm53b, Pichinde virus-AN3739 and Tacaribe virus-TRVL11573 was grown on BHK-21 cells; Junin virus-Candid#1 and SARS coronavirus-Tor2 were grown on Vero-E6 cells; Porcine respiratory and reproductive syndrome virus-SD-23983 was grown on MARC145 cells; Type II Feline coronavirus was grown on AK-D cells; Mouse hepatitis virus-A59 was grown on mouse 17Cl-1 cells; Influenza A virus-Udorn/307/72 was grown on MDCK cells; Influenza B virus-Beijing/1/87 was grown in embryonated chicken eggs; and Murine leukaemia virus Gag particles were expressed in SF9 cells using a recombinant baculovirus expression system [20].

### Sample Preparation and Imaging

Cryo-electron micrographs of *Coronaviridae* [8], *Arenaviridae* [21], and *Arteriviridae* [22], come from published studies. *Junin virus*, *Influenza A virus*, *Influenza B virus* and baculovirus-expressed *Murine leukaemia virus* Gag particles were purified by density gradient centrifugation and imaged as described previously [8]. Only near-focus images where the two phosphate rings were distinctly visible were used in this analysis (Fig. 1). Vesicles and GP vesicles used in this study were less than two times larger or smaller than their associated virus particles.

### Image Analysis

Micrographs were minimally processed using the ctfit module of EMAN before analysis to correct phase inversion effects [23]. The brightness of entire micrographs was normalized to a common mean value. Data was collected by selecting a rectangular region ∼80 Å wide and extending ∼100 Å above and below the low-density node of the membrane, with dimensions varying slightly depending on image pixel scale. Linear density traces (Fig. 1) were calculated by aligning images and averaging the signal from each ∼80 Å image row. Small errors in transect centering were corrected by ten cycles in which density traces were shifted by up to one pixel (3–5 Å) to find the alignment with the highest linear correlation to the group average for that round. Phosphate peak values were extracted from ∼25 Å regions as indicated in Fig. 1. A total of 27912 density traces were analyzed.

### Statistical Methods

For each virus a linear mixed-effects model was fitted by restricted maximum likelihood, assuming that the electron microscope signal intensity was determined by the membrane phosphate peaks (inner or outer) and particle type (virus, GP vesicle, or empty vesicle), including cross-effects. The sample region, particle and micrograph were included as nested random effects. Model fit was deemed acceptable by inspection of Q-Q plots. Significant differences in the mean signal value were subsequently evaluated using Tukey contrasts, to allow for multiple comparisons.
